# Heavy Metals and Health Risks Associated with Consumption of Herbal Plants Sold in a Major Urban Market in Southwest, Nigeria

**DOI:** 10.5696/2156-9614-11.31.210915

**Published:** 2021-08-17

**Authors:** Johnson A. Olusola, Oluwakemi B. Akintan, Harrison A. Erhenhi, Olagoke O. Osanyinlusi

**Affiliations:** 1 Institute of Ecology and Environmental Studies, Obafemi Awolowo University, Ile-Ife, Osun State, Nigeria; 2 Department of Geography and Planning Science, Ekiti State University, Ado Ekiti, Nigeria; 3 Department of Botany, Delta State University, Abraka, Delta State, Nigeria; 4 Jiann-Ping Hsu College of Public Health, Georgia Southern University, Statesboro, USA

**Keywords:** Herbal plants, Ado Ekiti, Health risk, Heavy metal

## Abstract

**Background.:**

Although herbal medicines play an important role as a source of medicine, concerns have been raised about the risks posed by consumption of these plants, especially if consumed above permissible levels.

**Objectives.:**

This study was undertaken to assess the level of exposure, toxicity and health risk associated with the consumption of herbal plants in Ado Ekiti urban market, Nigeria.

**Methods.:**

Ten commonly consumed herbal plants sold in Ado Ekiti urban market were subjected to heavy metal analysis. Health risk assessment was carried out to determine the estimated daily intake (EDI) of heavy metals, health risk index (HRI), target hazard quotient for non-carcinogenic risk and estimation of cancer risk (ECR).

**Results.:**

The EDIs for lead (Pb), nickel (Ni), chromium (Cr), copper (Cu) and magnesium (Mn) were above the upper tolerable daily intake reference for all studied plant species in both children and adults, an indication that herbal use poses a short-term to long-term health risk to consumers of these herbal products. The EDI for children was significantly lower compared to that of adults, indicating higher risks for adult consumers of these herbal products. The HRI in children for Pb (*Alstonia congensis*, *Terminalia avicennioides*, *Aframomum melegueta*, *Cymbopogon citratus* and *Napoleona vogelii*) were greater than 1; HRI in Cu and Mn also showed an unusually high concentration, an indication that long term exposure to the consumption of these herbal plants poses a serious health risk. The HRI in children and adults follows the order Mn > Cu > Ni > Pb > Cr; cadmium (Cd) was not detected in any of the herbal plants. The ECR for Pb, Ni and Cr present in the herbal plants for children ranged between 10^−6^ (low) to 10^−3^ (high), while the ECR for Pb, Ni and Cr for herbal plants for adults ranged between 10^−5^ (acceptable) to 10^−2^ (unacceptable). For both children and adults, there is a call for concern due to ECRs above the acceptable range; the consumption of these herbal plants poses a long-term cancer risk.

**Conclusions.:**

In both children and adults, ECRs for some of the herbal plants in the present study above the acceptable range present a risk to human health. The consumption of such herbal plants poses a long-term cancer risk.

**Competing Interests.:**

The authors declare no competing financial interests.

## Introduction

Herbal plants and medicine are heavily relied upon in Africa and globally. Herbal medicines, also known as botanical medicines or phytomedicines, refer to herbs, herbal materials, herbal preparations, and finished herbal products that contain parts of plants or other plant materials as active ingredients.^1^ Herbal medicines are currently widely used in complementary to orthodox medicine for the management of various illnesses.^2^ It is estimated that close to 80% of the population in low- and middle-income countries (LMIC) rely on herbal medicine for their basic healthcare needs.^3^ In Nigeria, 75% of the populace depends on herbal medicine.^4^ In most LMIC, the use of herbal remedies is relatively common, with a reported prevalence of 20–80% in the Caribbean,^5^,^6^ Trinidad,^7^,^8^ South Africa,^9^ and Nigeria.^10^,^11^

In Nigeria, despite the government's creation of a modern health care system, the patronage of herbal medicines and products remains high. For instance, Kayode and Sanni (2016) attributed the use of plant-derived medicine to their availability, cheap cost, and high efficacies with no appreciable side effects compared to modern medicine.^12^ On the other hand, it could be a result of poor or low efficacy of synthetic drugs.^13^ Modern health services have failed to meet the needs of the increasing population, especially in rural areas of Nigeria.^14^ The low level standard of living and lack of education create barriers to accessing adequate health services, so many resort to the use of traditional medicine.^14^

Several factors are confronting the development and implementation of herbal medicine in different parts of the world; some of these challenges include regulatory status, assessment of safety and efficacy, quality control, safety monitoring and inadequate or poor knowledge of traditional, complementary and alternative, and herbal medicines within national drug regulatory authorities.^15^ With regard to regulatory status, definitions and categorizations of herbal medicines differ among countries. For example a single medicinal plant could be categorized as food and medicine, medicinal diet or dietary supplement.^16^ Mixed herbal medicines may contain many different herbal plants. It is a challenge and sometimes practically impossible to isolate such herbal plants especially if there are different herbal plants mixed together.^15^ The quality and sources of materials used in the production of these herbal products determines their safety and efficacy.^16^,^17^ Correct identification of species of medicinal plants, special storage, and sanitation and cleaning methods for various materials are important requirements for quality control of herbal medicine materials.

Although herbal medicine plays an important role in traditional medicine, there have been concerns about the risks posed by these plants, especially if consumed above permissible limits. Odoh and Ajiboye, 2020 reported a high level of chromium (Cr), cobalt (Co) and lead (Pb) in selected herbal medicinal products consumed in Taraba State, Nigeria.^18^ Durodola *et al.*, (2019) also reported a high level of Co in herbal teas in Nigeria and concluded that it could pose a relative risk to consumers of such teas.^19^ Toxicities of these heavy metals have also been studied. For example, a high level of Pb has been associated with gastrointestinal irritation, loss of appetite and weight, sleeplessness, fatigue and headache.^20^,^21^ Other studies have reported the presence of cadmium (Cd) in herbal medicine.^22^,^23^ Cadmium consumption beyond its permissible level could lead to injury to the human respiratory system and irritation of the stomach which is a precursor to vomiting and diarrhea. Chronic exposure to Cd which is highly carcinogenic, could lead to lung and prostate cancer.^24^,^25^

**Abbreviations**
*ECR*: Estimation of cancer risk
*EDI*: Estimated daily intake
*HRI*: Health risk index

A high level of nickel (Ni) has been detected in some traditional medicines. Saeed, 2010, found Ni to be in the range of 0.2–56.3 μg/g in a group of branded herbal products in Pakistan.^23^ Hina, 2020, determined Ni to be in the range of 0.48–76.97 μg/g in a group of selected herbal products available in a local market in Karachi city, Pakistani.^26^ Nickel above its permissible level has been associated with dermatitis of the fingers, hands and forearms, respiratory distress, and a high risk of lung and nasal cancer from inhalation.^27^,^28^

Nickel is a major cause of genotoxicity, hematotoxicity, teratogenicity, immunogenicity and carcinogenicity.^29^,^30^ Chromium has been detected in many studies to be highly toxic.^19^,^31^,^32^,^18^ Chromium toxicity leads to allergic responses like urticaria.^33^,^34^ If ingested beyond acceptable limits, exposure to Cr could lead to anemia, damage to the male reproductive system, gastrointestinal irritation and heart-related diseases like cardiovascular and respiratory challenges.^35^,^36^,^37^ Guertin, 2004 reported that trivalent chromium is considered an essential trace element, while hexavalent chromium is carcinogenic in humans which could lead to stomach tumors.^33^

The indiscriminate, irresponsible or non-regulated use of herbal medicines may put the health of users at risk because of high levels of toxicity.^38^–^41^ The consumption of herbal products which is common in Africa could cause serious health risks. An assessment of the heavy metal content of these herbal drugs should be considered to determine their concentrations in plant materials to protect public health. When humans are exposed to moderate concentrations of these elements, either beneficial or toxic effects can occur, depending on their composition in the substance consumed.^32^ The aim of the present study was to assess the level of exposure, toxicity and health risk associated with the consumption of herbal plants bought from Ado Ekiti urban market for consumption by adults and children in Ado Ekiti, southwest Nigeria.

## Methods

In order to identify the most common herbal plants sold at Ado Ekiti urban market in southwest Nigeria, local market vendors were asked to identify the name of the plants in the local dialect (*Table 1*)*.* In total, 10 commonly consumed herb plants were randomly selected. Their medicinal uses are summarized in Table 1.

**Table 1:** Herbal Plants and Therapeutic Uses

**Botanical name**	**Local name**	**Part used**	**Used for**
*Sorghum bicolor*	Poroporo	Leaves	Blood supplement, acute stomachache, painful and irregular menstruation
*Senna fistula*	Opaeyin	Leaves	Treatment for back pain, antiviral, antidiabetic and antiulcerogenic
*Alstonia congensis*	Ahun	Leaves	Anti-diarrheal
*Terminalia avicennioides*	Idi	Leaves	Anti-diarrheal
*Aframomum melegueta*	Ewe atare	Leaves	Anti-malarial
*Cymbopogon citratus*	Beresi	Leaves	Anti-malarial
*Hibiscus acetosella*	Ewe ite	Leaves	Dysentery, cough, piles, convulsion, chest pain, snake or scorpion bite, stomachache, rheumatism
*Napoleona vogelii*	Atoo	Leaves	Bone setting
*Bambusa vulgaris*	Ewe oparun	Leaves	Analgesic. Effective against sexually transmitted diseases. Immune booster
*Theobroma cacao*	Epo koko	Leaves	Hemorrhoids, torso pain (e.g. constipation, irregular menstruation, obesity)

### Pretreatment and digestion of herbal plants

Glassware and containers were washed and rinsed with distilled water. Glassware was later soaked in nitric acid (HNO_3_) for 24h, cleaned thoroughly with distilled water, and dried to ensure that no cross-contamination occurred from the preparation process. Samples were washed and air-dried for one week at room temperature in a dust-free environment, then ground with a porcelain mortar and pestle into fine particles. The herbal plant samples were further air dried and ground to pass through 0.15-mm sieve. One (1) g of the sieved samples was weighed into a digestion tube and 10 mL of concentrated HNO_3_ was added and allowed to soak for 15 minutes. The sample was digested and heated at 25°C until frothing stopped and the HNO_3_ almost evaporated. Five (5) mL of concentrated perchloric acid (HClO_4_) was added and heating continued until the sample turned light in color. It was allowed to cool for some time, distilled water was later added and the sample filtered into a 100-mL volumetric flask and made up to the mark with distilled water.

Blank solutions were prepared using the samples and heavy metals were determined using a Buck scientific atomic absorption spectrometer (AAS) (Model: 210VGP) at appropriate wavelengths for each metal and detection limit. Heavy metals analysis was carried out at the Faculty of Agricultural Science laboratory, Ekiti State University, Ado Ekiti. Quantification of metals was based upon calibration curves of standard solutions of metals. Blanks were included in each batch for analysis and the certified reference standards were used to evaluate the accuracy of the analytical method. The blank determination was applied when the blank analysis gives results with a non-zero standard deviation. Wavelength and detection limits of the various heavy metals using blank calibration are shown in Table 2.

**Table 2:** Calibration Information

**Element**	**Wavelength (m)**	**Detection limit (LoD)**
Ni	232.0	0.05
Mn	279.5	0.005
Cd	228.9	0.01
Pb	283.3	0.08
Cr	357.9	0.04
Cu	324.8	0.01

### Health risk assessment

A human health risk assessment (HRA) is a characterization of the potential adverse health effects as a result of exposures to various environmental hazards.^42^ The HRA identifies and measures a hazard, determine possible routes of exposure (ingestion or dermal contact) and uses that information to calculate a numerical value to represent the potential risk.^43^

In the health risk assessment, heavy metals are classified as either cancer causing or non-cancer causing. Non-cancer-causing heavy metals are assumed to have a threshold; a dose below which no adverse health effects will be observed where an essential part of the dose-response portion of a risk assessment includes the use of a reference dose (*RfD*). Cancer-causing heavy metals do not have an effective threshold. This assumption implies that there is a risk of developing cancer with exposures at low doses and, therefore, there is no safe threshold for exposure to carcinogenic chemicals. Cancer causing elements are expressed by their cancer potency factor.^43^

The following variables constitute the health risk calculation for this study: estimated daily intake of heavy metal (EDI), health risk index (HRI), target hazard quotient (THQ) for non-carcinogenic risk, and estimation of cancer risk (ECR).

The EDI of a heavy metal through ingestion is given by Equation 1:

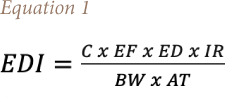



Where EDI is the estimated daily intake of heavy metal in the herbal plant (mg/kg/day), EF is the exposure frequency (350 days/year), ED is the exposure duration (average age of 7 years for children and 30 years for adults in Nigeria), IR is the ingestion rate of herbal drug (IR is assumed to be 0.25 L/day and 0.75 L/day for children and adults),^44^ C is the concentration of heavy metals in herbal plants (mg/kg), BW is body weight which is assumed to be 30 kg and 60 kg for an average child and adult in Nigeria, and AT is the averaging time based on life expectancy (years).

Average life expectancy (AT) is equal to exposure duration (ED) and the average life expectancy of a typical Nigerian was considered to be 55 years.^45^

The HRI was calculated using Equation 2:

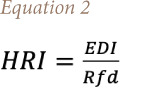



Where EDI is estimated daily intake of heavy metals from herbal plants, and Rfd is the oral reference dose for non-carcinogenic risk (Pb, Cd, Cu, Mn, Cr and Ni values are 0.0035, 0.001, 0.040, 0.14, 1.5 and 0.020 mg/kg/day respectively).^46^

The THQ can be generated from the hazard index (HI) and is given by Equation 3:

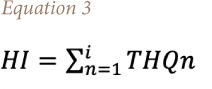



The ECR was calculated using Equation 4:

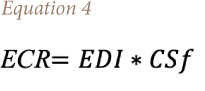



Where EDI is the estimated daily intake of a heavy metal; *CSf* is the ingestion slope factor (Pb=0.0085 and Ni=0.84).^47^–^49^ A cancer risk index ranging between 10^−6^ (1 in 1 000 000) and 10^−4^ (1 in 10 000) shows the predicted lifetime risks for cancer-causing agents. Chemicals with risk factors lower than 10^−5^ (1 in 10 000) are not considered to be chemicals of further concern.^50^,^51^

## Results

Table 4 and 5 show the estimated daily intake of heavy metal doses in the herbal plants ingested by children and adults. Estimated daily intake is a function of the frequency, duration and body weight of the persons exposed to the herbal plants; for this study a body weight of 30 kg and 60 kg was used for children and adults in Nigeria, with an exposure frequency of 350 days/year, exposure duration (7 years and 30 years for children and adult in Nigeria), while the ingestion rate of herbal plants in Nigeria was assumed to be 0.25 L/day and 0.75 L/day for children and adults. The EDI for Pb, Ni, Cr, Cu and Mn were above the upper tolerable daily intake reference for all studied plant species in both children and adults as shown in Table 4 and 5.

**Figure 1:**
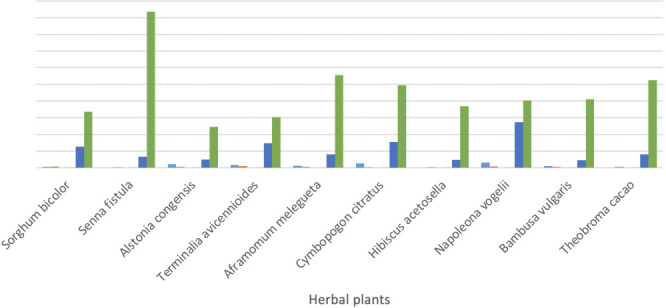
Non Carcinogenic Health Risk Index for children

Figure 1 shows that the HRIs for children for Pb (*Alstonia congensis, Terminalia avicennioides, Aframomum melegueta, Cymbopogon citratus* and *Napoleona vogelii)* were greater than 1 and the HRIs for Cu and Mn showed an unusually high HRI, an indication that long-term exposure to the consumption of these herbal plants poses a serious health risk. An HRI value greater than 1 indicates that consumption of the herbal plants poses a health risk, while an HRI lower than 1 is an indication that the consumption of the herbal product poses no health risk.

**Figure 2:**
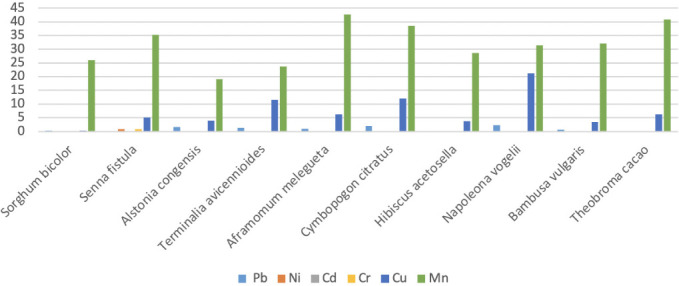
Non-carcinogenic Health Risk Index in adults

Figure 2 showing HR for adult; HRI followed the order Mn > Cu > Ni > Pb > Cr; Cd was not detected in any of the herbal plants.

Table 6 is a function of the estimation of the cancer risk (ECR) obtained from the various herbal products (all studied plant species). Cancer risk index ranging between 10^−6^ (1 in 1 000 000) and 10^−4^ (1 in 10,000) shows a predictable lifetime risks for cancer causing agents. Chemicals with risk factors less than 10^−6^ (1 in 1 000 000) may not be treated as chemicals for further concern.^49^,^50^ The ECR for Pb, Ni and Cr present in the herbal plants for children ranged between 10^−6^ (low) to 10^−3^ (high) while the ECR for Pb, Ni and Cr for herbal plants for adult ranged between 10^−5^ (acceptable) to 10^−2^ (unacceptable).^50^,^51^

## Discussion

The maximum residual limit (MRL) is a function of the maximum quantity of heavy metal residue in plants that will not pose any form of health risk, and above which represents a health risk to consumers. There were variations in MRI as shown in Table 3; in most of the herbal plants, Pb was within the MRI except *Cymbopogon citratus* and *Napoleona vogelii* which had levels above the MRI, although such levels may be tolerated. All the herbal plants were within the MRL for Ni; all but *Sorghum bicolor, Aframomum melegueta* and *Hibiscus acetosella* had Cr levels above the MRLs, which is a call for concern. MRLs vary from country to country and therefore may not be used for health risk assessment due to variations in consumption frequencies, dosage differences and average body weight.^31^

**Table 3:** Heavy Metal Concentrations in Selected Herbal Plants (mg/kg)

**Sample**	**Pb**	**Ni**	**Cd**	**Cr**	**Cu**	**Mn**
*Sorghum bicolor*	0.0200	0.2200	ND	0.0600	6.7000	62.5600
*Senna fistula*	ND	0.0800	ND	0.3000	3.4500	84.600
*Alstonia congensis*	0.1000	0.1200	ND	0.1000	2.6800	45.7000
*Terminalia avicennioides*	0.0800	0.2300	ND	0.5200	7.9000	56.8000
*Aframomum melegueta*	0.0600	0.1400	ND	0.0800	4.2500	102.4000
*Cymbopogon citratus*	0.1200	0.0800	ND	0.2000	8.2000	92.5000
*Hibiscus acetosella*	ND	0.3200	ND	0.0200	2.5600	68.9000
*Napoleona vogelii*	0.1400	0.2200	ND	0.3500	14.5500	75.4000
*Bambusa vulgaris*	0.0400	0.1200	ND	0.1800	2.4000	76.9000
*Theobroma cacao*	ND	0.0500	ND	0.0800	4.3000	98.2000
MRLs	0.1	0.6	NA	0.05	0.1	0.26

Abbreviations: NA, not applicable; MRL, maximum residual limits

**Table 4:** Estimated Daily Intake of Heavy Metals for Children (mg/kg (bw/day)

**Sample**	**Pb**	**Ni**	**Cd**	**Cr**	**Cu**	**Mn**
*Sorghum bicolor*	0.0012	0.0128	0.0000	0.0035	0.3908	3.6493
*Senna fistula*	0.0000	0.0047	0.0000	0.0175	0.2013	4.9350
*Alstonia congensis*	0.0058	0.0070	0.0000	0.0058	0.1563	2.6660
*Terminalia*	0.0047	0.0134	0.0000	0.0303	0.4608	3.3130
*avicennioides*						
*Aframomum melegueta*	0.0035	0.0082	0.0000	0.0047	0.2479	5.9733
*Cymbopogon citratus*	0.0070	0.0047	0.0000	0.0117	0.4783	5.3958
*Hibiscus acetosella*	0.0000	0.0187	0.0000	0.0012	0.1493	4.0192
*Napoleona vogelii*	0.0082	0.0128	0.0000	0.0204	0.8488	4.3983
*Bambusa vulgaris*	0.0023	0.0070	0.0000	0.0105	0.1400	4.4858
*Theobroma cacao*	0.0000	0.0030	0.0000	0.0047	0.2508	5.7283
Upper tolerable daily intake limit						
CA HP (mg/kg/day	3.33* 10^−4^	NA	1.00*10^−4^	3 33*10^−4^	NA	NA
FAO/WHO (mg/kg/day	1.67*10^−3^	1.00*10^−3^	3.33*10^−5^	8.33*10^−4^	5.00*10^−2^	4.33*10^−3^

*Abbreviations:* CA HP, Canadian upper tolerable daily intake reference limits for herbal plants (mg/kg bw/day)^52^; FAO/WHO, Food and Agricultural Organization/World Health Organization (mg/kg bw/day)^53^; NA- not applicable (upper tolerance limit of the heavy metal not available).

**Table 5:** Estimated Daily Intake of Heavy Metals for Adults (mg/kg (bw/day)

**Sample**	**Pb**	**Ni**	**Cd**	**Cr**	**Cu**	**Mn**
*Sorghum bicolor*	0.0075	0.0825	0.0000	0.0225	2.5125	23.4600
*Senna fistula*	0.0000	0.0300	0.0000	0.1125	1.2934	65.4500
*Alstonia congensis*	0.0375	0.0450	0.0000	0.0375	1.0050	17.1375
*Terminalia avicennioides*	0.0300	0.0863	0.0000	0.1950	2.9625	21.3000
*Aframomum melegueta*	0.0225	0.0525	0.0000	0.0300	1.5936	38.8400
*Cymbopogon citratus*	0.0450	0.0300	0.0000	0.0750	3.0750	34.6875
*Hibiscus acetosella*	0.0000	0.0120	0.0000	0.0075	0.9600	25.8375
*Napoleona vogelii*	0.0525	0.0825	0.0000	0.1313	5.4563	28.2750
*Bambusa vulgaris*	0.0150	0.0450	0.0000	0.0675	0.9000	28.8375
*Theobroma cacao*	0.0000	0.0375	0.0000	0.0300	1.6125	36.8250
Mean	0.0800	0.1580	0.0000	0.1890	5.6990	76.3960
SD	0.0432	0.0857	0.0000	0.1581	3.7919	18.4191
SEM	0.0165	0.0271	0.000	0.0500	1.1991	5.8246
Upper tolerable daily intake limit						
CA HP (mg/kg/day	3.33^*^10^−4^	NA	1.00^*^10^−4^	3.33^*^10^−4^	NA	NA
FAO/WHO (mg/kg/day	1.67^*^10^−3^	1.00^*^10^−3^	3.33^*^10^−5^	8.33^*^10^−4^	5.00^*^10^−2^	4.33^*^10^−3^

Abbreviations: CA HP, Canadian upper tolerable daily intake reference limits for herbal plants in mg/kg bw/day^52^; FAO/WHO, Food and Agricultural Organization/World Health Organization mg/kg bw/day^53^; NA- not applicable (upper tolerance limit of the heavy metal not available); SD, standard deviation; SE, standard error.

**Table 6:** Estimation of Cancer Risk

	**Children**			**Adults**		

**Sample**	**Pb**	**Ni**	**Cr**	**Pb**	**Ni**	**Cr**
*Sorghum bicolor*	1.1912^*^10^−6^	1.3684^*^10^−6^	2.2272^*^10^−4^	3.4773^*^10^−5^	3.78^*^10^−2^	6.1364^*^10^−3^
*Senna fistula*	0	4.9926^*^10^−4^	1.1136^*^10^−3^	0	1.37^*^10^−2^	3.0700^*^10^−2^
*Alstonia congensis*	6.307^*^ 10^−4^	7.4836^*^10^−4^	3.6909^*^10^−4^	1.7386^*^10^−4^	2.06^*^ 10^−2^	1.023^*^10^−2^
*Terminalia avicennioides*	5.0521 ^*^10^−6^	1.4326^*^10^−3^	1.9282^*^10^−3^	1.3909^*^10^−4^	3.954^*^ 10^−2^	5.3200^*^10^−2^
*Aframomum melegueta*	3.7864^*^10^−6^	8.7665^*^10^−4^	2.9909^*^10^−4^	1.0432^*^10^−4^	2.45^*^ 10^−2^	8.1820^*^10^−3^
*Cymbopogon citratus*	7.5727^*^10^−6^	5.0247^*^10^−4^	7.4455^*^10^−4^	2.0863^*^10^−4^	1.37^*^10^−2^	2.0500^*^10^−2^
*Hibiscus acetosella*	0	1.992^*^ 10^−3^	7.6364^*^10^−3^	0	5.4982^*^10^−3^	2.0455^*^10^−3^
*Napoleona vogelii*	8.8385^*^10^−6^	1.3684^*^10^−3^	1.2982^*^10^−3^	2.4341 ^*^10^−4^	3.78^*^10^−2^	3.5400^*^10^−2^
*Bambusa vulgaris*	2.5206^*^10^−6^	7.4836^*^10^−4^	6.6818^*^10^−4^	6.9545^*^10^−5^	2.062^*^ 10^−2^	1.8400^*^10^−2^
*Theobroma cacao*	0	3.2073^*^10^−4^	2.9909^*^10^−4^	0	1.695^*^ 10^−2^	8.1820^*^10^−3^

As shown in Tables 4 and 5, EDIs were high, an indication that herbal medicines pose short- to long-term health risks to consumers in Ado Ekiti, Nigeria. Studies have been carried out on the effects of exposure of heavy metals beyond tolerable limits. For instance, Pb exposure has been linked to gastrointestinal irritation and neuron toxicity in children and adults,^52^ and Cr ingestion beyond tolerable levels has been shown to promote carcinogenicity in stomach ulcers.^36^,^37^

Comparatively, EDIs for children were significantly lower than those of adults (*Tables 4 and 5*)*,* thus raising a major concern for adult consumers of these herbal products. This is a clarion call to the Nigerian National Food and Drug Administration and Control to take the lead in ensuring that standards are laid out for administering herbal medicines so that herbal plants associated with adverse human toxicological effects are more easily identified.

In the present study, the HRI followed the order Mn > Cu > Ni > Pb > Cr in Figure 2; Cd was not detected in any of the herbal plants. The HRI for non-carcinogenic metals indicates a long-term exposure impact for the various heavy metals. Excess Cu has been linked to hypotension, vomiting, hematemesis, coma, jaundice and necrosis of the tubular cells,^54^ it has also been associated with dilatation of the central veins and leads to congestion of glomeruli and necrosis of tubular cells in the kidneys.^55^ Beyond permissible levels, Mn contents have been linked to neurological disorders.^56^ In both children and adults, high ECRs were found for Pb, Ni and Cr (*Table 6*)*,* indicating that the consumption of the studied herbal plants poses a long-term cancer risk.

## Conclusions

The present study analyzed the health risks associated with the presence of heavy metals and consumption of herbal plants sold in Ado Ekiti urban market, southwest, Nigeria. The EDIs for Pb, Ni, Cr, Cu and Mn in the study were above the upper tolerable daily intake reference for all studied plant species in both children and adults, an indication that consumption poses a short- to long-term health risk to consumers of these herbal products. Comparatively, EDIs for children were significantly lower compared to those of adults, thus raising a major concern for adult consumers of these herbal products. For Pb, the HRI in children for *Alstonia congensis, Terminalia avicennioides, Aframomum melegueta, Cymbopogon citratus* and *Napoleona vogelii* was greater than 1 and the HRI or Cu and Mn also showed unusually high concentrations. The HRI in children and adults follows the order Mn > Cu > Ni > Pb > Cr. The ECR for Pb, Ni and Cr present in the herbal plants for children ranged between 10^−6^ (low) to 10^−3^ (high), while the ECR for Pb, Ni and Cr in herbal plants for adults ranged between 10^−5^ (acceptable) to 10^−2^ (highly unacceptable). In both children and adults, there is a call for concern due to ECRs above the acceptable range; the consumption of such herbal plants poses long-term cancer risk. There is a need for education on the dangers posed by consumption of herbal medicines in this area in order to protect human health. It is recommended that the NAFDAC should take the lead in ensuring that standards are laid out for herbal plants and products so that those associated with toxicological effects can be more easily identified.
